# Multiscale Deepspectra Network: Detection of Pyrethroid Pesticide Residues on the Hami Melon

**DOI:** 10.3390/foods12091742

**Published:** 2023-04-22

**Authors:** Guowei Yu, Huihui Li, Yujie Li, Yating Hu, Gang Wang, Benxue Ma, Huting Wang

**Affiliations:** 1 College of Mechanical and Electrical Engineering, Shihezi University, Shihezi 832003, China; ygw986119533@163.com (G.Y.); lyj_shzdx@163.com (Y.L.); hyt_0207@163.com (Y.H.); mrwanggang520@163.com (G.W.); xgb@shzu.edu.cn (H.W.); 2 Analysis and Testing Center, Xinjiang Academy of Agricultural and Reclamation Sciences, Shihezi 832000, China; huihuili189@126.com; 3 Food Quality Supervision and Testing Center (Shihezi), Ministry of Agriculture and Rural Affairs, Shihezi 832000, China; 4 Key Laboratory of Northwest Agricultural Equipment, Ministry of Agriculture and Rural Affairs, Shihezi 832003, China; 5 Engineering Research Center for Production Mechanization of Oasis Characteristic Cash Crop, Ministry of Education, Shihezi 832003, China

**Keywords:** visible/near-infrared spectroscopy, deep learning, pesticide residues, spectral analysis, Hami melon

## Abstract

The problem of pyrethroid residues has become a topical issue, posing a potential food safety concern. Pyrethroid pesticides are widely used to prevent and combat pests in Hami melon cultivation. Due to its high sensitivity and accuracy, gas chromatography (GC) is used most frequently for detecting pyrethroid pesticide residues. However, GC has a high cost and complex operation. This study proposed a deep-learning approach based on the one-dimensional convolutional neural network (1D-CNN), named Deepspectra network, to detect pesticide residues on the Hami melon based on visible/near-infrared (380–1140 nm) spectroscopy. Three combinations of convolution kernels were compared in the single-scale Deepspectra network. The convolution group of “5 × 1” and “3 × 1” kernels obtained a better overall performance. The multiscale Deepspectra network was compared to three single-scale Deepspectra networks on the preprocessing spectral data and obtained better results. The coefficient of determination ($R^{2}$) for lambda-cyhalothrin and beta-cypermethrin was 0.758 and 0.835, respectively. The residual predictive deviation (RPD) for lambda-cyhalothrin and beta-cypermethrin was 2.033 and 2.460, respectively. The Deepspectra networks were compared with two conventional regression models: partial least square regression (PLSR) and support vector regression (SVR). The results showed that the multiscale Deepspectra network outperformed the other models. It was found that the multiscale Deepspectra network could be a novel approach for the quantitative estimation of pyrethroid pesticide residues on the Hami melon. These findings can also provide an effective strategy for spectral analysis.

## 1. Introduction

Pyrethroid pesticides are widely used to prevent and combat pests due to their stability and high effectiveness, but they cause pesticide residue pollution problems in agricultural products worldwide [1]. The Chinese Standard (NY/T 427—2016) [2] limits the residue content of pyrethroid pesticides on muskmelons. Hami melon, a species of thick rind muskmelon, is the product of geographical indications in Xinjiang [3]. Its pulp is sweet and nutritious with a high reputation in the domestic and international markets. Hami melon is susceptible to pests during cultivation, and farmers often use pyrethroid pesticides for pest prevention. The excessive use of pyrethroid pesticides will cause residues on the Hami melon surface and in the soil, contaminating the fruit. Pyrethroid pesticide residues have become a topical issue in society and pose a potential safety concern for agricultural products. Therefore, finding a rapid and nondestructive method to detect pyrethroid pesticide residues on the Hami melon is necessary.

Gas chromatography (GC) is the most frequently used method to detect pyrethroid pesticide residues due to its high sensitivity and accuracy [4]. However, it requires sample pretreatment, and the pretreatment and detection steps are complex and costly [5]. Visible/near-infrared (Vis/NIR) spectroscopy, which is a potential detection technique for pesticide residues, has the advantages of being rapid, nondestructive, convenient, and low-cost [6]. Some scholars conducted a qualitative analysis of pesticide residues in fruits and vegetables based on Vis/NIR spectroscopy. Ndung’u et al. [7] used short-wave NIR spectroscopy (750–900 nm) to rapidly screen for pesticides (a formulation of beta-cyfluthrin and chloropyriphos, another formulation of metalaxyl and mancozeb) in spinach. Using principal component data, the shallow-learning approaches of support vector machines (SVM), random forest (RF), and artificial neural networks (ANN) achieved a perfect accuracy of 100%. Chen et al. [8] proposed the contrastive principal component analysis to reduce the feature dimension of near-infrared spectral data (900–1700 nm). The results showed that this method could classify the fruits (apple and pear) as with or without chlorpyrifos residues. For the discrimination of chlorpyrifos residue levels (mixing ratio of pesticide and water as 1:0, 1:200, 1:500, 1:800, and 1:1000), a back propagation neural network (BPNN) based on Vis/NIR spectroscopy (300–2500 nm) was proposed and obtained a better test accuracy of 96.67% [9]. Nazarloo et al. [10] used Vis/NIR spectroscopy (400–1050 nm) with partial least squares discriminant analysis (PLS-DA) to identify the safe and unsafe levels of profenofos residues. The model accuracy of the prediction set was 91.66%.

Most qualitative analyses of pesticide residues obtained good accuracy. Moreover, Vis/NIR spectroscopy also has the potential to quantitatively estimate pesticide residues. Yazici et al. [11] used NIR spectroscopy (900–2500 nm) and partial least squares regression (PLSR) to detect compound pesticide residues (boscalid and pyraclostrobin) in strawberries. The residual predictive deviation for boscalid and pyraclostrobin was 2.28 and 2.31, respectively. Nazarloo et al. [12] detected profenofos residues on tomatoes using Vis/NIR spectroscopy (350–1100 nm) and the ANN model. After spectral feature extraction with the successive prediction algorithm (SPA), the model performance was the best with a coefficient of determination of 0.982 and a root mean square error of 0.166. The above prediction results were admissible.

Previous studies mainly focused on detecting pesticide residues in fruits and vegetables by combining Vis/NIR spectral information with shallow-learning methods. As the deep-learning approach evolved, further studies indicated that Vis/NIR spectral analysis using the end-to-end deep-learning networks could improve the model accuracy in discriminating the pesticide residues of fruits and vegetables. A single-scale, one-dimensional convolutional neural network (1D-CNN) was proposed to recognize pesticide residues (lambda-cyhalothrin, trichlorfon, phoxim, and mixtures of trichlorfon and phoxim) on garlic chive leaves, achieving a better accuracy of 97.9% [13]. 1D-CNN models using multiscale convolution were proposed to identify the types and levels of pesticide residues on the Hami melon [14,15]. The test results showed that the multiscale convolution networks provided a better model performance than the single-scale networks. The 1D-CNN model performed well in the qualitative analysis of pesticide residues. However, the use of deep-learning networks for quantitatively estimating pesticide residues in fruits and vegetables has yet to be investigated.

The objectives of this study were (1) to explore the feasibility of Vis/NIR spectroscopy for the detection of pyrethroid pesticide residues on the Hami melon; (2) to establish Deepspectra networks based on the 1D-CNN and evaluate the impact of convolution kernel combination and architecture on the Deepspectra networks; and (3) to investigate the potential of Deepspectra networks in the spectral analysis compared to conventional regression models.

## 2. Materials and Methods

### 2.1. Sample Preparation

A total of 140 Hami melons (Xizhoumi) were purchased from a local agricultural product trading center in Shihezi, Xinjiang, China. We chose two pyrethroid pesticides (lambda-cyhalothrin and beta-cypermethrin) as the research object, and they were purchased from a local agricultural material market in Shihezi, Xinjiang, China. The pesticide specifications are shown in Table 1. All Hami melons were stored at 25 °C and a relative humidity of 30% until sample preparation. The pesticide solution was prepared by mixing lambda-cyhalothrin and beta-cypermethrin with water in ratios of 1:200, 1:400, and 1:800. There were 35 Hami melon samples in each group under three different ratios of the pesticide solution. The remaining Hami melons were sprayed with clean water as a control group. All samples were placed in the laboratory for ten hours until spectral data acquisition.

### 2.2. Spectral Data Acquisition and Preprocessing

#### 2.2.1. Instrument and Software for Acquisition

**Figure 1:**
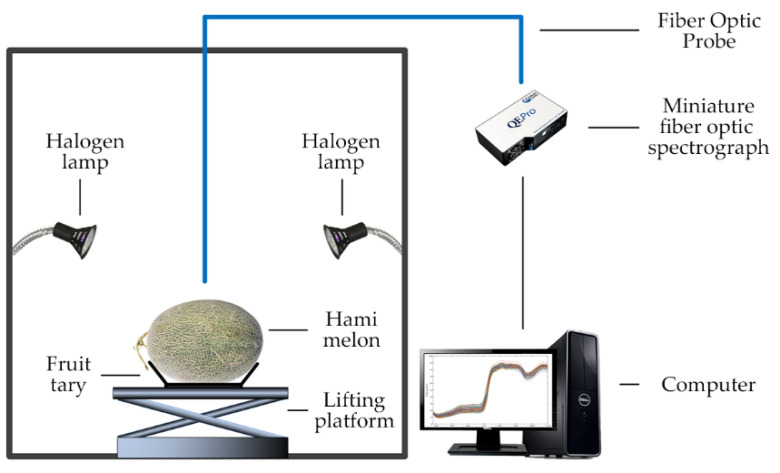
Visible/near-infrared spectroscopy system [14].

Figure 1 shows the visible/near-infrared spectroscopy system used in this study, including a miniature fiber optic spectrograph with a spectral resolution of 0.69 nm, a fiber optic probe, a light source consisting of two halogen lamps, a fruit tray, a lifting platform, and a computer with a spectrometer operating software. The wavelength range of the spectrum used in this study was 380–1140 nm.

Table 2 shows the specifications of the main instruments and software for spectral data acquisition. The integration time, moving average width, and average number of scans were 0.1 s, 4, and 10, respectively. Before sample spectrum spectral data acquisition, the initial spectrum (R_initial_) was calibrated into reflectance spectrum (R_calibration_) by using the white and dark references, as shown in Equation (1). The dark reference (R_dark_) was obtained by turning off the light source. The white reference (R_white_) was obtained by using a white Teflon bar when the light source was turned on.

(1) $$R_{\mathrm{calibration}}=\frac{R_{\mathrm{initial}}-R_{\mathrm{calibration}}}{R_{\mathrm{white}}-R_{\mathrm{dark}}}$$

#### 2.2.2. Spectral Acquisition Position

**Figure 2:**
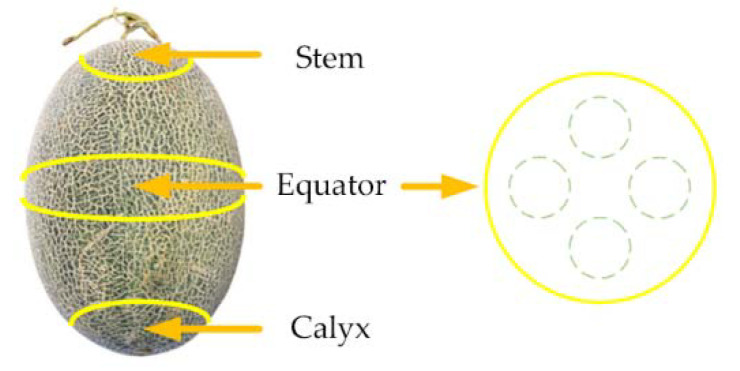
Spectral acquisition position [16].

To obtain the representative spectrum as the sample spectrum, we selected the spectral acquisition position using the method proposed by Hu et al. [16], as shown in Figure 2. There were three positions (stem, equator, and calyx) for each Hami melon. Then, four regions with an interval angle of 90° were marked at the equator position. Therefore, we acquired four spectra from each Hami melon and recorded its average spectrum as the sample spectral data.

#### 2.2.3. Spectral Data Preprocessing

To enhance the spectral resolution and sensitivity, the first-order derivative (1st D), as a widely-used preprocessing method, was used to remove background interference, eliminate baseline drift, and separate superposed peaks [17]. In this study, the derivative was computed with the Savitsky–Golay convolution. The number of points in the filter was 5. The order of the polynomial was 2.

### 2.3. Reference Measurement of the Pesticide Residue Contents

The reference measurement was performed after spectral data acquisition. The reference values of the pesticide residue contents were measured in the Food Quality Supervision and Testing Center (Shihezi), Ministry of Agriculture and Rural Affairs. The measurement procedure was consistent with Yu et al. [15]. (1) Standard preparation: The standard mixture intermediate and working solutions were prepared in n-hexane at a concentration of 20.0 mg/mL and 1.0 mg/mL, respectively. The solutions were stored in brown reagent bottles at 4 °C and placed at room temperature before use. (2) Sample Preparation: The pulps and rinds of each Hami melon were cut into samples with a thickness of approximately 1.50 cm, and the samples were crushed in a food processor. Then, the treated samples were transferred to the marked sample bottles. They were stored at −18 °C and placed at room temperature before measurement. A QuEChERS (quick, easy, cheap, effective, rugged, and safe) method was used for sample preprocessing, including extraction and clean-up, according to the British Standard (BS EN 15662:2008) [18]. (3) Extraction: A 7.5 g amount of the crushed sample was weighed with an electronic balance and was transferred to a 50 mL centrifuge tube. Then, 15 mL of acetonitrile was added. The mixture was vortexed at a speed of 3000 r/min with a vortex shaker for 40 s. After homogenization for 1 min, 5 g of NaCl was added to the mixture and again vortexed at a speed of 3000 r/min for 40 s. Subsequently, the tubes were centrifuged with a high-speed centrifuge at a speed of 7000 r/min for 5 min to separate the two layers. An 8 mL volume of the supernatant was removed. (4) Clean-up: An 8 mL volume of the supernatant was transferred to a 15 mL QuEChERS clean-up centrifuge tube. Then, the mixture was vortexed at a speed of 3000 r/min for 40 s and centrifuged at a speed of 7000 r/min for 5 min. A 4 mL volume of the supernatant was transferred to a glass tube and evaporated to dryness with a nitrogen evaporator. Finally, the extract was redissolved in 2 mL of n-hexane. Table 3 shows the specifications of the main instruments and reagents for the standard preparation, sample preparation, extraction, and clean-up. (5) GC measurement: A gas chromatograph with a micro electron capture detector (GC–μECD) was used for reference measurement of the pesticide residue contents according to the Chinese Standard (NY/T 761—2008) [19]. The GC–μECD conditions are shown in Table 4.

### 2.4. Deepspectra Network Implementation

#### 2.4.1. Environment

The computations were performed on a Lenovo computer with a Windows 10 (64-bit) operating system and an Intel (R) Core (TM) I7-8700 @3.20 GHz CPU. All Deepspectra networks were implemented on PyTorch 1.13.1 framework using Python 3.7.3 in Spyder IDE 3.3.3.

#### 2.4.2. Architecture

**Figure 3:**
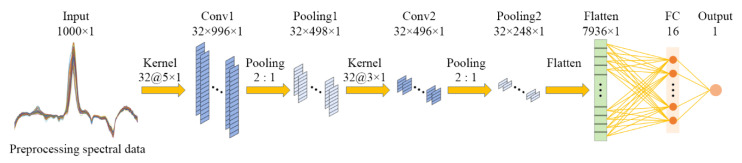
Architecture of the single-scale Deepspectra network. 1000 × 1 is the input of the network, which is the preprocessing spectral data with a length of 1000 and a dimension of 1; Kernel 32@d × 1 is 32 convolution kernels of the size d × 1; 2:1 is the size of the max pooling kernel; 32 × l × 1 is the size of the output feature map of the network layer, which is the depth × length × width; 7936 × 1 is the size of the output feature map of the flattened layer, which is the length × width; 16 and 1 are the number of the neurons in the network layer.

A Deepspectra network based on the 1D-CNN was developed. The architecture of a typical CNN is structured as a series of layers, including convolution (Conv), pooling, flattened (Flatten), and fully connected (FC) layers [20]. The input of the network was the preprocessing spectral data. The output of the network was the object character. The capability of the CNN model to capture features can improve by stacking the convolution and pooling layers. As a one-dimensional signal, the Vis/NIR spectral data had a low dimension and density, so we stacked the stage of convolution and pooling layers one time. The single-scale Deepspectra network had an input layer, two convolution layers, two max-pooling layers, a flattened layer, a fully connected layer, and an output layer, as shown in Figure 3.

**Figure 4:**
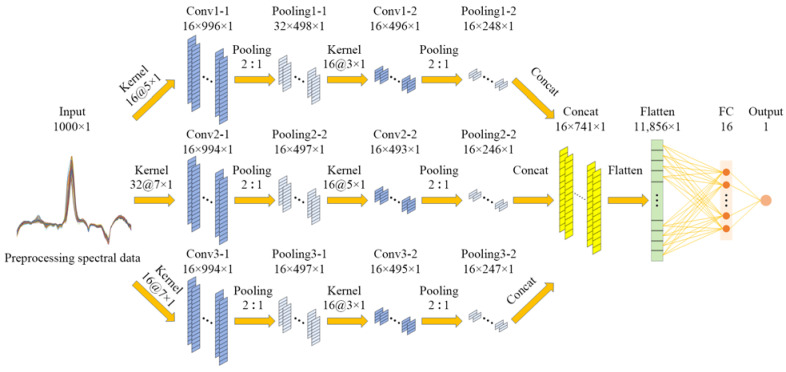
Architecture of the multiscale Deepspectra network. 1000 × 1 is the input of the network, which is the preprocessing spectral data with a length of 1000 and a dimension of 1; Kernel 16@d × 1 is 16 n convolution kernels of the size d × 1; 2:1 is the size of the max pooling kernel; 16 × l × 1 is the size of the output feature map of the network layer, which is the depth × length × width; 11,856 × 1 is the size of the output feature map of the flattened layer, which is the length × width; 16 and 1 are the number of the neurons in the network layer.

Increasing the network depth is the most straightforward method to improve the performance of the Deepspectra network, but it renders the enlarged network more prone to overfitting [21]. The multichannel convolution provides an effective solution. A Deepspectra network based on the parallel convolution architecture was proposed for quantitative spectral analysis [22]. To evaluate the effect of the multichannel convolution architecture on model performance, we designed the multiscale Deepspectra network. The multiscale Deepspectra network had an input layer, three parallel convolution channels, a concatenation layer (Concat), a flattened layer, a fully connected layer, and an output layer, as shown in Figure 4. Moreover, two convolutional and two max-pooling layers were in each convolution channel. The concatenation layer was used for deep-feature fusion after multichannel convolution, and the concatenation axis was the length, shown in Equation (2) to (4).

(2) $$y_{c}^{d\times l\times w}=y_{1}^{d\times l\times w}+y_{2}^{d\times l\times w}+y_{3}^{d\times l\times w}$$

(3) $$l_{c}=l_{1}+l_{2}+l_{3}$$

(4) $$d_{c}=d_{1}=d_{2}=d_{3},\;w_{c}=w_{1}=w_{2}=w_{3}=1$$

where $y_{c}$ is the output feature map of the concatenation layer; $y_{1}$, $y_{2}$, and $y_{3}$ are the output feature map of three convolution channels; and *d*, *l*, and *w* are the depth, length, and width of the feature map, respectively.

#### 2.4.3. Hyperparameters

Generally, a large convolution kernel has a large receptive field and obtains better global features. However, using multiple large convolution kernels can lead to an explosion of parameters [23]. Therefore, we used a larger kernel for the first convolution layer and a smaller kernel for the second convolution layer. The convolution channels 1, 2, and 3 used the combination of convolution kernels as “7 × 1 and 5 × 1”, “7 × 1 and 3 × 1”, and “5 × 1 and 3 × 1”, respectively. To control network parameters, 32 and 16 kernels were used in the single-scale and multiscale Deepspectra networks, respectively. The pooling mode was max pooling. The padding of convolution and pooling was valid. The stride of the sliding window in the convolution and pooling layers was 1 and 2, respectively. We chose the rectified linear unit (ReLU) as the activation function in the convolution layers, and it was the most widely used and effective [24]. The fully connected and output layers had 16 and 1 neurons, respectively. The linear was the activation function in the output layer. The mean square error (MSE) was the loss function, shown in Equation (5).

(5) $$\mathrm{MSE}=\frac{\sum_{i=1}^{n}{\left(y_{i,\;\;\;\mathrm{actual}}-y_{i,\;\;\;\mathrm{predicted}}\right)}^{2}}{n}$$

where $y_{i,\;\mathrm{actual}}$ is the reference values of the pesticide residue contents in the *i*-th Hami melon sample; $y_{i,\;\mathrm{predicted}}$ is the predicted values of the pesticide residue contents in the *i*-th Hami melon sample; and *n* is the number of Hami melon samples in the corresponding dataset.

The adaptive moment estimation (Adam) was used to optimize model training. The learning rate was 0.005. The remaining parameters of the Adam optimizer were default. Considering the exponential scale of $2^{n}$ and the small size of the sample dataset, we chose a small batch size of 16. Moreover, batch normalization was added after each convolution layer. Furthermore, it was also used after the flatten layer to replace the dropout method, which could effectively accelerate Deepspectra network training and avoid overfitting [25]. The max epochs were set to 100. To obtain the best model, we chose the weight for modeling when the loss of the validation set was minimal.

### 2.5. Conventional Regression Models

For Vis/NIR spectral analysis, partial least square regression (PLSR) and support vector regression (SVR) were the most used as the linear and nonlinear multivariate quantitative correction methods [26]. We chose the best latent variables (LVs) to establish the PLSR model. The radial basis function (RBF) was used as the kernel function in the SVR model, and its hyperparameters of the penalty coefficient (c) and kernel function parameter (g) were optimized with grid search (GS). The mathematical computing software MATLAB (R2016b, MathWorks Inc., Natick, MA, USA) was used to establish the conventional regression models.

### 2.6. Model Evaluation

Four parameters are often used for the evaluation of model performance, including the coefficient of determination ($R^{2}$) and the root mean square error (RMSE) for calibration ($R_{c}^{2}$, RMSEC), validation ($R_{v}^{2}$, RMSEV), and prediction ($R_{p}^{2}$, RMSEP), which are shown in Equations (6) and (7) [27]. In addition, the residual predictive deviation (RPD) of the prediction set is also an evaluation parameter, which is shown in Equation (8). Generally, a regression model with better performance has higher values of $R^{2}$ and lower values of RMSE [28]. In RPD, values of 1.5–2.0 are initiatory for prediction, whereas values of 2.0–2.5 make an admissible prediction, values of 2.5–3.0 are suitable for prediction, and values of >3.0 are sufficient for application [29].

(6) $$R^{2}=1-\frac{\sum_{i=1}^{n}{\left(y_{i,\;\;\;\mathrm{actual}}-y_{i,\;\;\;\mathrm{predicted}}\right)}^{2}}{\sum_{i=1}^{n}{\left(y_{i,\;\;\;\mathrm{actual}}-{\bar{y}}_{\mathrm{actual}}\right)}^{2}}$$

(7) $$\mathrm{RMSE}=\sqrt{\frac{\sum_{i=1}^{n}{\left(y_{i,\;\;\;\mathrm{actual}}-y_{i,\;\;\;\mathrm{predicted}}\right)}^{2}}{n}}$$

(8) $$\mathrm{RPD}=\frac{1}{\sqrt{1-R_{p}^{2}}}$$

where $y_{i,\;\mathrm{actual}}$ is the reference values of the pesticide residue contents in the *i*-th Hami melon sample; $y_{i,\;\mathrm{predicted}}$ is the predicted values of the pesticide residue contents in the *i*-th Hami melon sample; ${\bar{y}}_{\mathrm{actual}}$ is the mean reference value of all Hami melon samples; and n is the number of Hami melon samples in the corresponding dataset.

## 3. Results

### 3.1. Statistics of the Reference Values

The reference values of five samples were abnormal, so one hundred samples were used for this study. The maximum and minimum lambda-cyhalothrin residue contents were 32.36 and 0.96 µg/g, respectively. The maximum and minimum beta-cypermethrin residue contents were 12.74 and 0.37 µg/g, respectively. The concentration fluctuation range of the pesticide residues was extensive. It was necessary to adopt an appropriate data division method to obtain an ideal dataset. Typically, 20% of the original sample set was used as a validation, 20% as a prediction, and 60% as a correction set [27]. Moreover, this study used an interval sampling method to divide the 100 samples [30]. Table 5 shows the statistics of the residue contents of lambda-cyhalothrin and beta-cypermethrin in the dataset. The residue contents of beta-cypermethrin were lower than those of lambda-cyhalothrin. The concentration fluctuation range of the calibration set covered entirely the range of the validation prediction set. In addition, the average values and standard deviation (SD) values of the three sets were close to each other. The statistical characteristics indicated that the sample division was reasonable [31].

### 3.2. Spectral Characteristics

**Figure 5:**
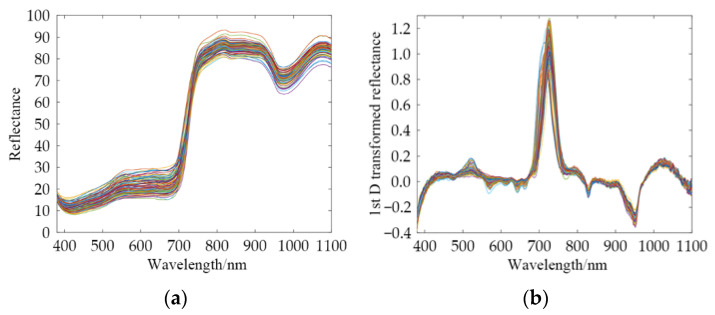
Vis/NIR diffuse reflection spectra of the pesticide residues on the Hami melon. (**a**) Raw spectra; (**b**) 1st D transformed spectra.

Figure 5 shows the Vis/NIR raw diffuse reflection spectra and 1st D transformed spectra of the pesticide residues on the Hami melon. The rind color of the Xizhoumi Hami melon is green due to chlorophylls. The bright color of the chlorophylls obscured other pigments [7]. There were two absorption peaks (410–430 and 670–680 nm) in the visible region (400–700 nm), which were associated with the absorption bands of the chlorophylls [16]. The weak absorbance peak at 830–840 nm was associated with the third overtone of the C–H functional group [32]. The strong absorbance peak at 970–980 nm was associated with water [33]. Similar tendencies in the raw spectra demonstrated that each sample had similar components. The different spectral reflectance suggested differences in the pesticide residues. We needed to analyze the spectral data further using the Deepspectra networks.

### 3.3. Impact of the Convolution Kernel Combination and Architecture on the Deepspectra Networks

Table 6 shows the results of the Deepspectra networks for detecting lambda-cyhalothrin and beta-cypermethrin residues. Three combinations of convolution kernels were compared. For all convolution combinations, the $R_{c}^{2}$ was over 0.990, the $R_{v}^{2}$ was over 0.810, the RMSEC was low, and the RMSEV was higher than the RMSEC. The results showed that all single-scale Deepspectra networks obtained better training results without overfitting. For the detection of the lambda-cyhalothrin residues, when the combination of convolution kernels was 5 × 1 and 3 × 1, the prediction results were the best: the $R_{p}^{2}$ was 0.725, the RMSEP was 4.606, and the RPD was 1.909. The single-scale Deepspectra network configured with the 5 × 1 and 3 × 1 convolution kernel had an initiatory prediction performance. The prediction result of the beta-cypermethrin residues was better than that of the lambda-cyhalothrin residues. The RPD of three Deepspectra networks was over 2.200. It indicated that the prediction was admissible. The convolution kernel combination of 7 × 1 and 3 × 1 obtained the best results: the $R_{p}^{2}$ was 0.814, the RMSEP was 1.484, and the RPD was 2.320.

For the detection of the lambda-cyhalothrin and beta-cypermethrin residues, the best combination of convolution kernels in the single-scale Deepspectra network was different. The above results show that the single combination of convolution kernels could not adapt to detecting different pesticide residues. The impact of the multichannel convolution architecture on the Deepspectra network was investigated. For the detection of the lambda-cyhalothrin residues, the $R_{p}^{2}$ was over 0.750, and the RPD was over 2.000. The model prediction performance was admissible. For the detection of the lambda-cyhalothrin residues, the $R_{p}^{2}$ was over 0.830, and the RPD was close to 2.500. The results showed that the multichannel convolution architecture could improve the model performance. Moreover, the detection of the lambda-cyhalothrin residues was better than that of the beta-cypermethrin residues due to lower RMSE, higher $R^{2}$, and admissible RPD in the calibration, validation, and prediction sets.

### 3.4. Comparison of the Deepspectra Networks with Conventional Regression Models

**Figure 6:**
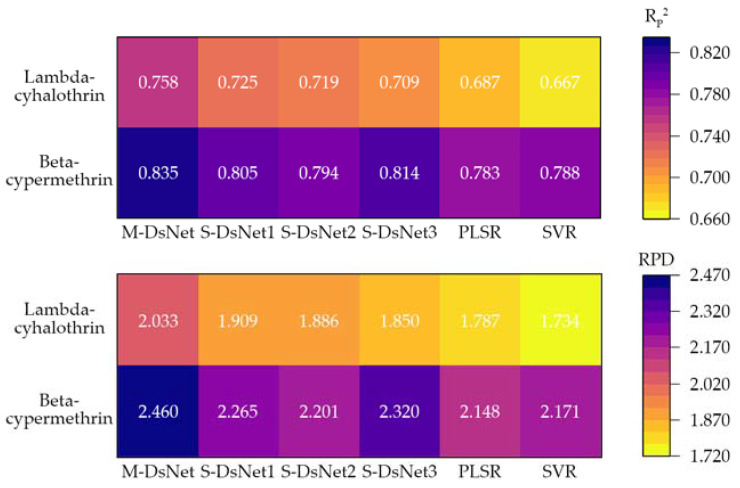
Results of the Deepspectra networks and conventional regression models. The single-scale Deepspectra network configured convolution kernels of 5 × 1 and 3 × 1, 7 × 1 and 5 × 1, and 7 × 1 and 3 × 1 were named S-DsNet1, S-DsNet2, and S-DsNet3, respectively; the multiscale Deepspectra network was named M-DsNet.

Figure 6 shows the results of the Deepspectra networks and conventional regression models on the prediction set. The Deepspectra networks provided better performance than the conventional regression models. The $R_{p}^{2}$ and RPD of PLSR and SVR were much lower than the best performance of the Deepspectra networks. Compared with PLSR in detecting the lambda-cyhalothrin residues, the $R_{p}^{2}$ and RPD of the multiscale Deepspectra network were improved by 10.33% and 13.77%, respectively. Moreover, only the performance of the multiscale Deepspectra network was admissible. The model performance of the worst Deepspectra network was also higher than PLSR and SVR.

The detection results of the beta-cypermethrin residues on conventional regression models were better than those of the lambda-cyhalothrin residues. It corresponded to the Deepspectra networks. The $R_{p}^{2}$ of PLSR and SVR was improved but was less than 0.800. The RPD of PLSR and SVR was over 2.000, which showed that the prediction performance was admissible. However, it was also much lower than the performance of the multiscale Deepspectra network. Compared with SVR in detecting the lambda-cyhalothrin residues, the $R_{p}^{2}$ and RPD of the multiscale Deepspectra network were improved by 5.96% and 13.31%, respectively. Three single-scale Deepspectra networks also outperformed the conventional regression models.

## 4. Discussion

This study proposed Vis/NIR (380–1140 nm) spectroscopy coupled with Deepspectra networks to detect two pyrethroid pesticide residues (lambda-cyhalothrin and beta-cypermethrin) on the Hami melon. The results showed that the single-scale Deepspectra network stacked with the 1D stage of the convolution and pooling layers was successfully used for Vis/NIR spectral analysis. It was consistent with Tian et al. [24] and Chen et al. [34]. We further studied the impact of convolution kernel combinations on the Deepspectra networks. We found that the optimal convolution kernel combination in detecting different pesticide residues was different. The RPD of the single-scale Deepspectra network was less than 2.0 for the detection of the lambda-cyhalothrin residues. It showed that the deep features captured with the single-channel convolution were insufficient to detect two pesticides.

To improve the ability of the Deepspectra network to capture multilevel features, we proposed the multiscale Deepspectra network incorporating three-channel convolution. The prediction results indicated that the multiscale Deepspectra network provided improved performance. The combination of convolution kernels used in each channel was different. It allowed the Deepspectra network to capture different scales of the local features. The multiscale Deepspectra network learned patterns from limited spectral deep features through concatenation mode. This corresponded to a previous study that suggested that an end-to-end deep-learning approach based on the Inception module performs better [22]. A review of spectral and deep-learning-based quality evaluation of food and agricultural products also suggested that the multichannel convolution effectively improved the performance of deep networks [35].

In addition, all Deepspectra networks outperformed the conventional regression models. Significantly, the model performance of the multiscale Deepspectra network was much higher than PLSR and SVR. However, the RPD of the multiscale Deepspectra network was at least 2.5. It suggested that the prediction performance needed to be improved for the application. We will improve the Deepspectra network architecture and optimize the hyperparameters so that its RPD can reach 2.5 or over 3.0. A large dataset could also effectively improve the performance of deep-learning models during training time [36]. The small amount of data used in this study may be another reason for the unsatisfactory performance of the Deepspectra networks.

No matter which Deepspectra network we chose, detecting the beta-cypermethrin residues obtained a better result. It may be due to the dispersion of the data set. The beta-cypermethrin dataset with a low SD of 3.41 had good stability. The stability of the dataset was also an essential factor in establishing a suitable model [30]. In addition, we need to consider the impact of individual differences on the Hami melon. More Vis/NIR spectral data of pesticide residues on the Hami melon need to be acquired to train the model to improve the robustness of the Deepspectra network in the future.

As mentioned above, end-to-end deep-learning approaches (take the multiscale Deepspectra network as a representative) have potential application values in the quantitative spectral analysis of pesticide residues. Sindhu et al. [37] also reported a similar point.

## 5. Conclusions

Deepspectra networks were designed to capture features from Vis/NIR spectra without dimensional reduction and feature extraction based on prior knowledge. We performed a Vis/NIR spectroscopy coupled with the multiscale Deepspectra network to detect lambda-cyhalothrin and beta-cypermethrin residues on Hami melon. Our findings can provide a theoretical basis and strategy for detecting pesticide residues on the large and thick rind fruit. In addition, the multiscale Deepspectra network included three parallel convolution channels to capture different global and local features, which looked promising for quantitative spectral analysis.

Extending the method’s scope and demonstrating its practical applicability in future studies will be critical. It puts forward higher requirements for the repeatability and adaptability of the model. On the other hand, the Deepspectra networks were end-to-end deep-learning approaches, and the feature extraction is performed in a ‘black box’. Therefore, it will also be interesting to explain and visualize the spectral depth feature extraction process.

## Figures and Tables

**Table 1:** The pesticide specifications.

Pesticide	Dosage Form	Active Ingredient Content	Manufacturing Company
Lambda-cyhalothrin	Microemulsion	2.5%	Shandong Caoda Chemical Co., Ltd., Heze, Shandong, China
Beta-cypermethrin	Emulsifiable concentrate	4.5%	Jinan Yinong Chemical Co., Ltd., Jinan, Shandong, China

**Table 2:** Specifications of the main instruments and software for spectral data acquisition.

Acquisition Instrument and Software	Version	Manufacturing Company
Miniature fiber optic spectrograph	QE Pro-FL	Ocean Insight, Inc., Dunedin, FL, USA
Fiber optic probe	QP600-2-VIS-NIROOS-00-5172-11	Ocean Insight, Inc., Dunedin, FL, USA
Halogen lamp	MR16	Signify (China) Investment Co., Ltd., Shanghai, China
Spectrometer operating software	OceanView 1.6.7	Ocean Insight, Inc., Dunedin, FL, USA

**Table 3:** Specifications of the main instruments and reagents for reference measurement of the pesticide residue contents.

Instrument/Reagent	Specification	Manufacturing Company
Electronic balance	BSA4202S-CW	Sartorius Inc., Gottingen, Germany
Vortex shaker	MS 3 Control	IKA Inc., Staufen, Germany
High-speed centrifuge	TG16-WS	Xiangyi Centrifuge Instrument Co., Ltd., Changsha, China
Nitrogen evaporator	N-EVAP-112	Organomation Associates, Inc., Burlington, VT, USA
GC–μECD	Agilent 7890A	Agilent Technologies Inc., Santa Clara, CA, USA
Certified pesticide standard solution	1000 mg/L and purities greater > 98.0%	Agro-Environmental Quality Supervision and Testing Center, Ministry of Agriculture and Rural Affairs, Tianjin, China
n-hexane	chromatographically pure CAS 110-54-3	Duksan Pure Chemicals Co., Ltd., Ansansi, Korea
Acetonitrile	chromatographically pure	ANPEL Laboratory Technologies (Shanghai) Inc., Shanghai, China
QuEChERS clean-up centrifuge tube	5982-0029 400.1 mg PSA, 400.1 mg C18 EC, 45.0 mg bulk carbograph, and 1199.8 mg magnesium sulfate (purity from 98.5% to 101.5%)	Agilent Technologies Inc., Santa Clara, CA, USA

**Table 4:** The GC–μECD conditions.

Parameters	Conditions
Analytical column	HP-5, 30 m × 0.320 mm × 0.25 μm
Injection volume	1 μL
Injection mode	Spitless
Carrier gas	Nitrogen, constant flow, 2.0 mL/min
Septum purge	3.0 mL/min
Makeup	60 mL/min
Inlet temperature	220 °C
Oven temperature	100 °C for 1 min then 15 °C/min to 190 °C and hold for 2 min then 6 °C/min to 280 °C and hold for 2 min
Detector temperature	320 °C

**Table 5:** The statistics of the reference values of the pesticide residue contents in the dataset.

Pesticides	Dataset	Number of Samples	Residue Contents/(µg/g)			
			Max	Min	Mean	SD
Lambda-cyhalothrin	Calibration set	60	32.36	0.96	10.91	8.83
	Validation set	20	31.79	1.22	11.32	9.03
	Prediction set	20	31.31	1.14	10.97	8.79
	Total samples	100	32.36	0.96	11.00	8.86
Beta-cypermethrin	Calibration set	60	12.74	0.37	3.72	3.37
	Validation set	20	12.21	0.46	3.84	3.47
	Prediction set	20	12.19	0.42	3.76	3.44
	Total samples	100	12.74	0.37	3.75	3.41

**Table 6:** Results of the Deepspectra networks with different combinations of convolution kernels.

Pesticides	Combination of Convolution Kernels	Calibration Set		Validation Set		Prediction Set		
		$R_{c}^{2}$	RMSEC	$R_{v}^{2}$	RMSEV	$R_{p}^{2}$	RMSEP	RPD
Lambda-cyhalothrin	7 × 1 and 5 × 1	0.997	0.490	0.823	3.795	0.719	4.661	1.886
	7 × 1 and 3 × 1	0.997	0.488	0.810	3.940	0.709	4.751	1.850
	5 × 1 and 3 × 1	0.996	0.471	0.828	3.742	0.725	4.606	1.909
	Multiscale	0.997	0.446	0.829	3.729	0.758	4.324	2.033
Beta-cypermethrin	7 × 1 and 5 × 1	0.996	0.222	0.828	1.441	0.794	1.565	2.201
	7 × 1 and 3 × 1	0.997	0.182	0.850	1.347	0.814	1.484	2.320
	5 × 1 and 3 × 1	0.997	0.190	0.832	1.426	0.805	1.520	2.265
	Multiscale	0.995	0.232	0.876	1.223	0.835	1.400	2.460

## Data Availability

The data presented in this study are available on request from the corresponding author.
